# Subconjunctival Orbital Fat Prolapse: A Case Report

**DOI:** 10.1055/s-0044-1793946

**Published:** 2024-11-21

**Authors:** Rajesh S. Powar, Soujanya M.

**Affiliations:** 1 Department of Plastic and Reconstructive Surgery, KLE University, Jawaharlal Nehru Medical College, Belgaum, Karnataka, India

**Keywords:** subconjuctival, orbital, fat prolapse, conjunctiva, orbital septum

## Abstract

Subconjunctival orbital fat prolapse is a benign condition where the orbital fat, which normally cushions the eye within its socket, protrudes or herniates through the conjunctiva, the clear membrane covering the sclera. It is a rare condition that occurs due to disruption of the orbital septum due to various causes. In this case study, we report the clinical findings, treatment, and insights gained from the diagnosis and management of a 59-year-old male patient with subconjunctival orbital fat prolapse.

## Introduction

Subconjunctival fat prolapse is a non-threatening rare condition characterized by the protrusion of orbital fat through the conjunctiva. This condition typically presents as a yellowish or white soft mass bulging from the nasal or temporal corners of the eye 1 2 often occurring due to age-related changes, trauma, or surgical procedures around the eye that disrupt the orbital septum. We aim to describe our experience with managing this rare pathology and discuss the current literature available on it.

## Case Report

**Fig. 1:**
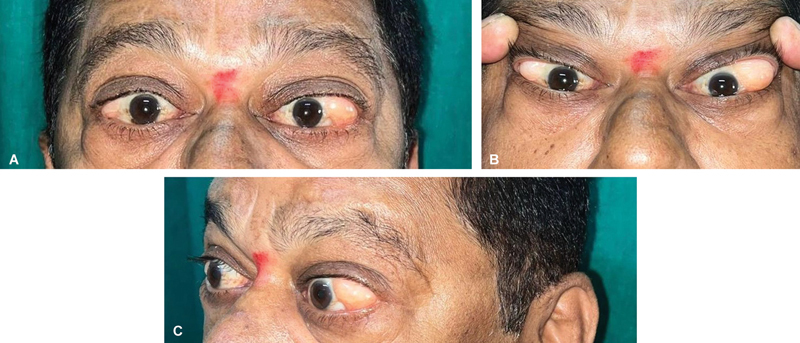
( **A** ) Preoperative swelling in both eyes. ( **B** ) Preoperative swelling in both eyes. ( **C** ) Preoperative swelling in both eyes (lateral view).

A 59-year-old man presented with a 9-year history of swelling in the outer aspect of both eyes, more pronounced in the left eye than the right. The swelling was insidious in onset and gradually progressive (see Fig. 1 ). He had no history of trauma, visual disturbances, associated pain, or discharge. The patient had a known history of ischemic heart disease (IHD) and underwent coronary angioplasty 10 years ago. There was no history of previous ophthalmic surgical interventions.

### Clinical Examination

General physical examination: The patient is moderately built and moderately nourished, conscious, cooperative, and well oriented to time, place, and person, with a weight of 78 kg, height of 174 cm, and body mass index (BMI) of 25.8 kg/m ^2^ .

### Local Examination

- Local examination revealed the following:
- A well-defined soft yellowish isolated mass in lateral aspect of both eyes with no continuity with nasojugal fat pad of the infraorbital region, lesion in the left eye measuring 2.0 × 1.5 cm and lesion in the right eye measuring 1.5 × 1.2 cm.
- Bilateral lesions were compressible, nontender, and well-defined anterior border with nonvisualization of the posterior border and no signs of inflammation.
- Visual acuity was 6/6 on both eyes.
- Fundoscopy was unremarkable.

A magnetic resonance imaging (MRI) scan of both orbits of the patient was conducted. The brain was examined in axial, sagittal, and coronal lanes with 5-mm contiguous slices.

## Magnetic Resonance Imaging Report

**Right orbit:** An elongated soft tissue, measuring approximately 1.4 × 0.4 cm along the right superolateral aspect of the orbit, which is continuous with the intraconal fat, was seen, suggesting herniation. There was no obvious movement of the lacrimal gland.

**Left orbit:** An elongated soft tissue, measuring approximately 2.0 × 0.8 cm along the left superolateral aspect of the orbit, which is continuous with the intraconal fat, was observed, suggesting herniation. There was no obvious movement of the lacrimal gland.

**Fig. 2:**
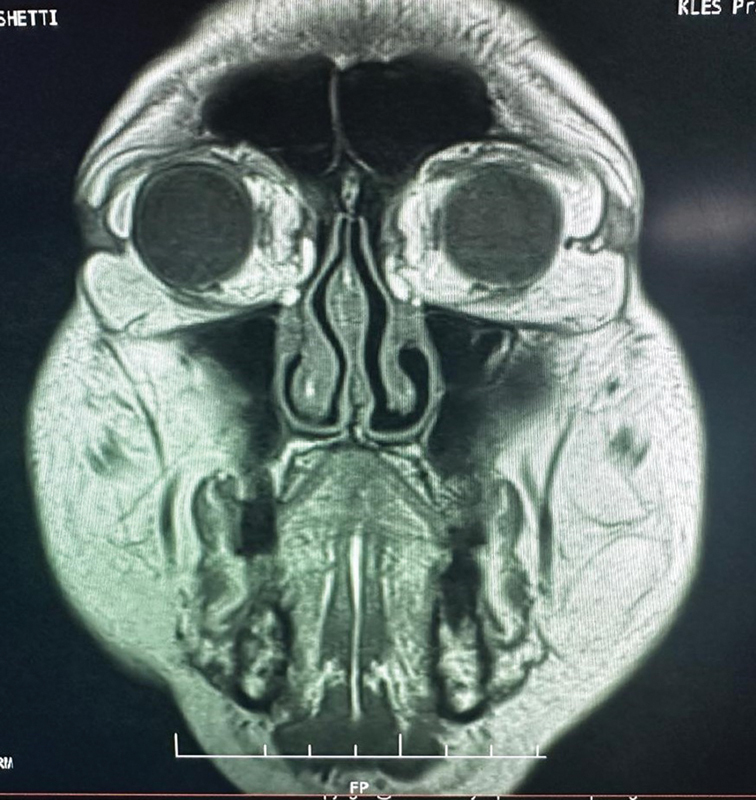
Magnetic resonance imaging.

The eyeballs, lens, and anterior and posterior segments of both the orbits were normal. Both the optic nerves, optic chiasma, optic tracts, extraocular recti, oblique muscles, and retrobulbar spaces on both sides appeared normal in the right and left orbits (see Fig. 2 ).

The diagnosis based on these findings was subconjuctival fat prolapse. The patient consented to surgical excision of the herniated fat. Considering the patient's history of angioplasty and ongoing clopidogrel medication, a cardiologist's consultation was obtained. Clopidogrel was discontinued 5 days prior to the surgery and restarted after 24 hours.

## Surgical Procedure

The patient was scheduled for single-staged bilateral transconjunctival excision of subconjunctival orbital fat.

**Fig. 3:**
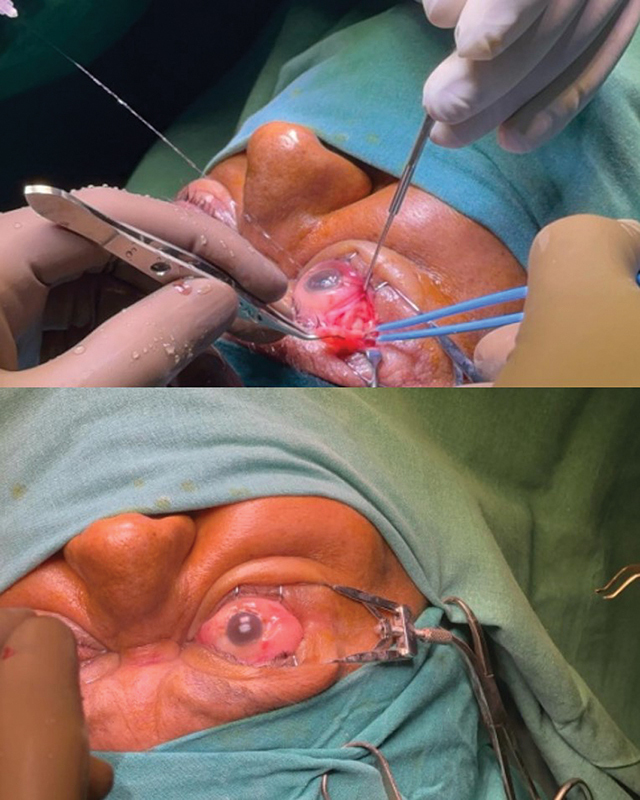
Intraoperative transconjunctival excision of herniated fat.

A peribulbar block was given in the bilateral infraorbital region using 10 mL of 2% lignocaine + hyaluronidase. Eye speculum and lid retractor were applied for adequate surgical field exposure. A 2-mm transconjunctival incision was placed over the right superotemporal region. Dissection was continued up to the identification of the lateral rectus and the orbital fat excised in total from the right eye. Incision was closed primarily using Nylon 10–0 suture (see Fig. 3 ). A similar procedure was performed for excision of subconjunctival orbital fat in the left eye in the same sitting.

Postoperatively, topical antibiotics and eye lubricants were started on the patient. On the first postoperative day, the patient exhibited moderate conjunctival congestion but no visual disturbances. The patient was subsequently monitored regularly as an outpatient.

**Fig. 4:**
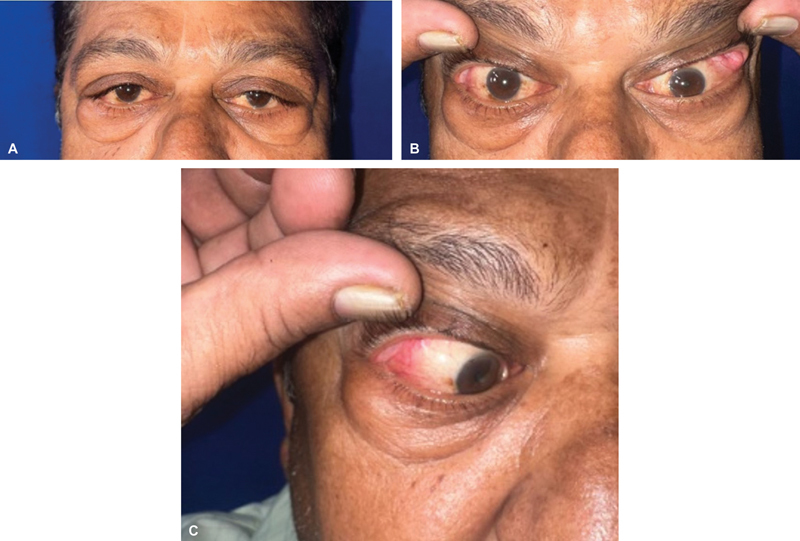
( **A** ) Postoperative result after 4 weeks. ( **B** ) Postoperative result after 4 weeks. ( **C** ) Postoperative result after 4 weeks.

Fig. 4 illustrates the cosmetic improvement of both eyes 4 weeks after surgery.

## Discussion

Subconjunctival fat prolapse is a rare, non-threatening condition characterized by the protrusion of orbital fat through the conjunctiva, typically due to age-related changes. 1 3 It is primarily a cosmetic concern and can be managed with observation or minor surgery if needed.

Subconjunctival fat prolapse often occurs due to age-related changes. As people age, the orbital septum (a fibrous membrane that helps contain the orbital fat) weakens, allowing the fat to push through and become visible. It can also result from trauma or surgical procedures around the eye, which may disrupt the orbital septum. 2

It is a visible, prominent, and soft mass in the conjunctiva, usually not associated with pain. Some patients may experience a sensation of fullness or mild irritation. The protrusion may cause concern for its appearance rather than physical symptoms. 4

Diagnosis is primarily clinical, based on the appearance of the mass and its characteristic location. An ophthalmologist can usually identify the condition through a routine eye examination. 5 Imaging studies such as an orbital computed tomography (CT) scan or MRI may be conducted to rule out other causes of orbital masses. 3

The differential diagnoses for subconjunctival fat prolapse include conjunctival dermolipoma, lymphoma, epidermoid cyst, and lacrimal gland prolapse. 1 2 6 But the primary differential diagnosis is conjunctival dermolipoma, a congenital benign lesion typically present at birth commonly that affects young women, with an average patient age of 22 years. 6 7 Conjunctival dermolipoma presents as a congenital soft or firm pinkish-white or pinkish-yellow mass with hairs on the surface, whereas subconjunctival fat prolapse appears as a soft, mobile yellowish mass. 2 Conjunctival dermolipoma is usually unilateral, immobile, and noncompressible. In imaging studies with CT and MRI, it appears as a crescent-shaped, fat-containing mass located in the temporal aspect of the orbit, distinct from the intraconal fat. 3 6 7

Subconjunctival fat prolapse is generally benign and does not require treatment if it is asymptomatic. However, if the prolapse causes significant discomfort or cosmetic concerns, treatment options may include surgical excision of the herniated fat. 7 8 9

Surgical intervention is necessary in the cases where the prolapse is bothersome. It is a minor surgical procedure that can be performed to reposition the fat and reinforce the orbital septum 9 Earlier studies, such as those by Siban et al, have shown that the recurrence rate after transconjunctival excision is very low (∼9%). 9

## Conclusion

Subconjunctival fat prolapse is a benign condition where orbital fat protrudes through the conjunctiva, usually due to age-related changes. Primarily a cosmetic issue, it may cause minor irritation or significant discomfort in some patients. Although rare, this condition can be effectively treated by a straightforward and safe transconjunctival excision.
